# Erratum to: ‘Draft genome sequence of Bacillus azotoformans MEV2011, a (Co-) denitrifying strain unable to grow with oxygen’

**DOI:** 10.1186/s40793-015-0057-2

**Published:** 2015-09-30

**Authors:** Maja Nielsen, Lars Schreiber, Kai Finster, Andreas Schramm

**Affiliations:** Section for Microbiology, Department of Bioscience, Aarhus University, Aarhus, Denmark; Center for Geomicrobiology, Department of Bioscience, Aarhus University, Aarhus, Denmark; Stellar Astrophysics Centre, Department of Physics and Astronomy, Aarhus University, Aarhus, Denmark

Unfortunately, the original version of this article [1] contained an error. The citation details was included incorrectly as 9:23. The correct citation is 10:4.
